# Molecular epidemiological investigation of G6PD deficiency in Yangjiang region, western Guangdong province

**DOI:** 10.3389/fgene.2023.1345537

**Published:** 2024-01-09

**Authors:** Hong-Feng Liang, Yan-Bin Cao, Fen Lin, Yi-Kang Yang, Yu-Wei Liao, Wei-Hao Ou, Jin-Ling Chen, Yan-Qing Zeng, Yu-Chan Huang, Guang-Kuan Zeng, Zhi-Xiao Chen, Jing-Wei Situ, Jin-Xiu Yao, Li-Ye Yang

**Affiliations:** ^1^ Precision Medical Lab Center, People’s Hospital of Yangjiang, Yangjiang, Guangdong, China; ^2^ Precision Medical Lab Center, Chaozhou Central Hospital, Chaozhou, Guangdong, China; ^3^ Institute of Medicine and Nursing, Hubei University of Medicine, Shiyan, Hubei, China; ^4^ Department of Laboratory Medicine, People’s Hospital of Yangjiang, Yangjiang, Guangdong, China; ^5^ Department of Transfusion, People’s Hospital of Yangjiang, Yangjiang, Guangdong, China

**Keywords:** G6PD deficiency, G6PD mutation, DNA sequence, China, gene variant

## Abstract

**Objectives:** The prevalence of G6PD deficiency has not been reported in Yangjiang, a western city in Guangdong province. This study aims to investigate the molecular characteristics of G6PD deficiency in this region.

**Methods:** Blood samples were collected from adults at a local hospital to screen for G6PD deficiency. The deficient samples were subjected to further analysis using PCR and reverse dot blot to determine the specific G6PD variants.

**Results:** Among the 3314 male subjects, 250 cases of G6PD deficiency were found using the G6PD enzyme quantitative assay, resulting in a prevalence of 7.54% (250/3314) in the Yangjiang region. The prevalence of G6PD deficiency in females was 3.42% (176/5145). Out of the 268 cases of G6PD deficiency tested for G6PD mutations, reverse dot blot identified 20 different G6PD variants. The most common G6PD variant was c.1388G>A (81/268), followed by c.1376G>T (48/268), c.95A>G (32/268), c.1024C>T (9/268), c.392G>T (7/268), and c.871G>A/c.1311C>T (6/268). It was observed that c.871G>A was always linked to the polymorphism of c.1311C>T in this population.

**Conclusion:** This investigation into G6PD deficiency in this area is expected to significantly improve our understanding of the prevalence and molecular characterization of this condition.

## 1 Introduction

Glucose-6-phosphate dehydrogenase (G6PD) deficiency is a widely prevalent condition found worldwide. Individuals with G6PD deficiency have increased susceptibility to oxidative stress, such as from fava beans and infections, which can lead to acute hemolytic anemia (Lee et al., 2022). Moreover, newborns with G6PD deficiency face a higher risk of developing jaundice, which can rapidly progress to bilirubin encephalopathy and cause permanent and catastrophic neurological damage known as kernicterus (Xu et al., 2021).

The G6PD gene is located at the Xq28 band and encodes the G6PD enzyme. It consists of 13 exons, 12 introns, and spans approximately 18 kb. G6PD deficiency is predominantly caused by genetic mutations following an X-linked incomplete recessive inheritance pattern. These mutations often give rise to polymorphic variants that exhibit reduced enzyme activity and disrupt protein folding (Pfeffer et al., 2022). Currently, more than 230 clinically relevant genetic variants have been identified (Pfeffer et al., 2022). The frequency of G6PD deficiency varies among different regions and ethnic populations (Lee et al., 2022; Pfeffer et al., 2022). In southern China, G6PD deficiency is prevalent (Lin et al., 2018). However, while data on the general population’s prevalence of G6PD deficiency is available, limited information exists regarding the range of G6PD variants in specific regions and ethnic groups (He et al., 2020).

Previous surveys have shown a higher occurrence of G6PD deficiency in Guangdong province (Yang et al., 2015; Lin et al., 2018; He et al., 2020). Nonetheless, the specific genotypes of G6PD variants have not been identified, and no large-scale survey has been conducted to investigate the molecular characteristics of G6PD deficiency in Yangjiang, a city situated in the western region of Guangdong province. Hence, the current study aims to determine the prevalence and molecular characterization of G6PD deficiency in Yangjiang, with the objective of enhancing our understanding of this condition in the region.

## 2 Material and methods

### 2.1 Study population and sample collection

The data for G6PD screening was collected from August 2018 to November 2022. The study included two groups: one group consisted of individuals visiting the People’s Hospital of Yangjiang for routine check-ups, while the other group comprised pregnant women attending the obstetrics department as outpatients for regular prenatal examinations. All participants were unaware of their G6PD status and underwent G6PD quantitative assays along with routine blood tests. Following the routine medical blood tests, the remaining blood samples were used for G6PD gene variant analysis. The study population consisted of 3,314 healthy local males and 5,145 pregnant females, aged between 15 and 50 years, who underwent G6PD enzyme quantitative assays. The study was approved by the Ethics Committee of the People’s Hospital of Yangjiang (NO. 20220069).

### 2.2 G6PD enzyme assay

The G6PD enzyme assay involved detecting the production rate of NADPH, which served as a quantitative method for assessing G6PD activity. The assays were conducted following the manufacturers' protocols (Beijing Antu Bioengineering Co., Ltd., China). In adults, a threshold of <1300 U/L was used to define G6PD deficiency (Tang et al., 2018).

### 2.3 Molecular analysis of G6PD gene variants

In individuals diagnosed with G6PD deficiency, whole blood collected with EDTA anticoagulants was stored in a biobank at −40°C. 268 cases of G6PD deficiency received gene analysis. The whole blood DNA extraction kit (Chaozhou Hybribio Co., Ltd.) was used to extract DNA according to the instructions of the kit, and then the DNA quantity and purity were tested (NanoDrop One, Thermo Fisher Scientific Co., Ltd.). Veriti^TM^ Dx 96-Well Thermal Cycler (Thermo Fisher Scientific) was used for PCR amplification, then, *G6PD* gene variant was detected by reverse dot blot method (Chaozhou Hybribio Co., Ltd.) for 13 common *G6PD* mutation types *c.1376G>T* (*G6PD* Canton), *c.1388G>A* (*G6PD* Kaiping), *c.95A>G* (*G6PD* Gaohe), *c.871 G>A* (*G6PD* Viangchan), *c.392G>T* (*G6PD* Qing Yan), *c.487G>A* (*G6PD* Mahidol), *c.493A>G* (*G6PD* Taipei), *c.592G>T* (*G6PD* Coimbra), *c.1004C > T* (G6PD Fushan), *c.1024C>T* (*G6PD* Chinese-5), *c.1360C > T* (*G6PD* Union), *c.1387C>T* (*G6PD* Keelung), *c.1381G>A* (*G6PD* Yunan) and one polymorphism *c.1311C>T* (Hu et al., 2015).

100 healthy cases (comprising both G6PD deficient and G6PD normal) were chosen randomly for G6PD gene analysis during routine check-ups, they were firstly tested for G6PD enzyme quantitative assay, and the gene sequencing method was described previously (Huang et al., 2023).

### 2.4 Statistical analysis

Statistical analysis in this study was carried out using SPSS 16.0 statistical software. Gene frequencies of the G6PD alleles were calculated using the Maximum Likelihood method. Hardy-Weinberg equilibrium was utilized to compare the observed and expected genotypes in the study. The distribution of various alleles responsible for G6PD deficiency in Yangjiang and other regions in China was compared using the chi-square test.

## 3 Results

In this study, 426 cases G6PD deficiency were identified by G6PD enzyme quantitative assay in 8459 adults, the prevalence of G6PD deficiency was 5.04% in this population, 250 cases of G6PD deficiency were found in males, the prevalence of G6PD deficiency was 7.54% (250/3314) in Yangjiang region, while the prevalence of G6PD deficiency in females was 3.42% (176/5145), it was lower than that of males.

**FIGURE 1:**
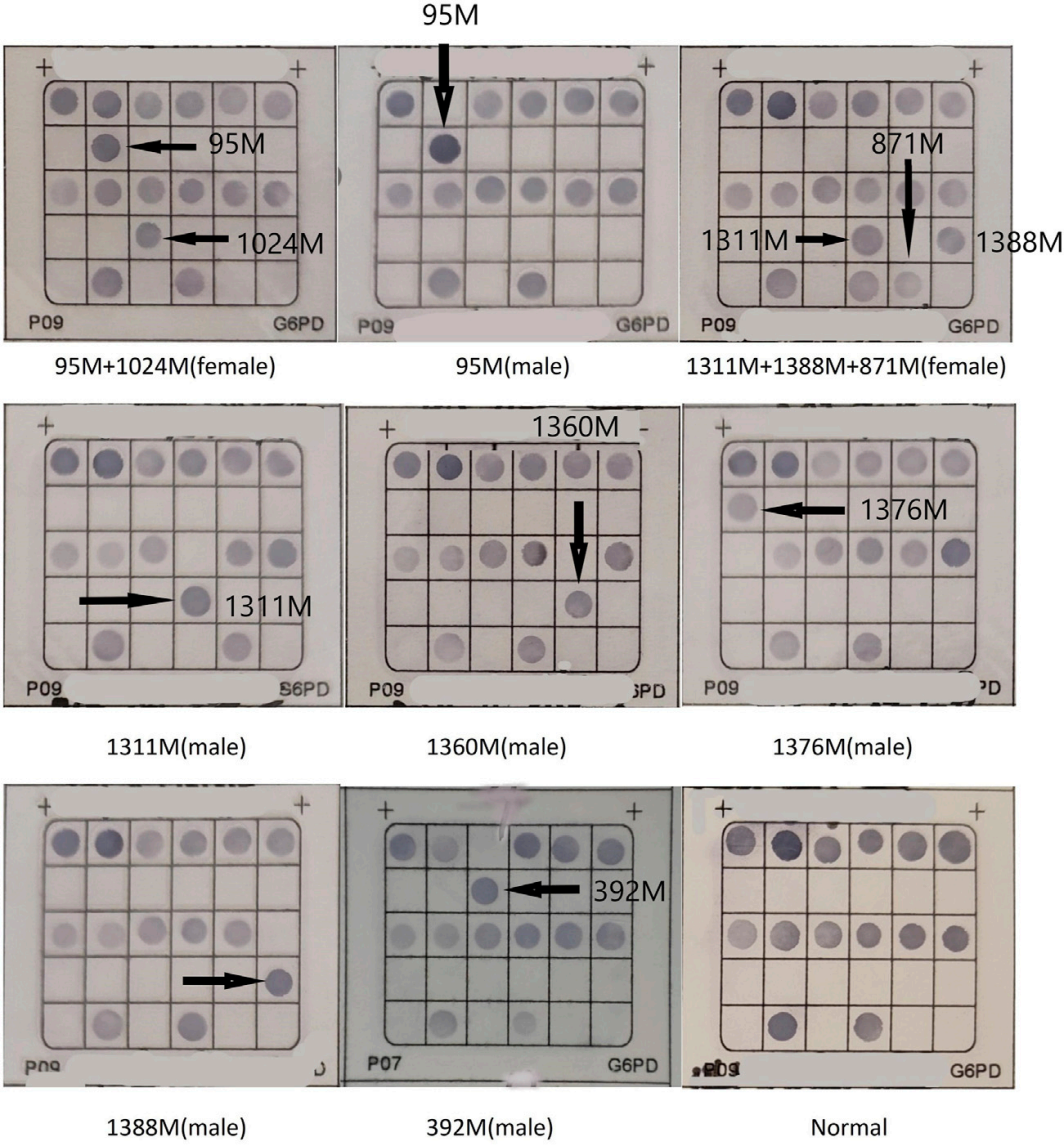
The hybridization results of G6PD gene chip. Locations of mutant probes are denoted M.

268 cases of G6PD deficiency were tested for G6PD mutations, 20 kinds of G6PD variants were identified by reverse dot blot. c.1388G>A (81/268) was the most common G6PD variant, followed by c.1376G>T (48/268), c.95A>G (32/268), c.1024C>T(9/268), c.392G>T(7/268), and c.871G>A/c.1311C>T (6/268). c.871G>A was always linked with polymorphism of c.1311C>T in this population (Figure 1; Table 1). 33 samples were not detected with G6PD mutations, the unidentified samples might be rare types not included in this detection kit (Table 1).

**TABLE 1:** G6PD genotypes distribution in 268 cases of G6PD deficiency.

Mutation	Hemizygote(n)	Heterozygote(n)	Homozygote(n)	Total (n)	Percentage %
unidentified	9	0	24	33	12.32
c.95A>G	15	17	0	32	11.94
c.392G>T	3	4	0	7	2.61
c.1024C>T	4	5	0	9	3.36
c.1311C>T	7	10	1	18	6.72
c.1360C>T	2	1	0	3	1.12
c.1376G>T	24	22	2	48	17.91
c.1388G>A	35	45	1	81	30.23
c.871G>A/c.1311C>T	2	4	0	6	2.24
c.392G>T/c.1388G>A	0	2	0	2	0.75
c.392G>T/c.1311C>T	0	1	0	1	0.37
c.1376G>T/c.95A>G	0	1	0	1	0.37
c.95A>G/c.1024C>T	0	1	0	1	0.37
c.95A>G/c.1311C>T	0	3	0	3	1.12
c.95A>G/c.1388G>A	0	1	0	1	0.37
c.1376G>T/c.392G>T	0	1	0	1	0.37
c.1376G>T/c.1311C>T	0	7	0	7	2.61
c.1376G>T/c.1388G>A	0	3	0	3	1.12
c.1311C>T/c.1388G>A	0	9	0	9	3.36
c.1024C>T/c.1311C>T/c.871G>A	0	1	0	1	0.37
c.1311C>T/c.1388G>A/c.871G>A	0	1	0	1	0.37
Total	101	139	28	268	100

100 healthy adults (44 males and 56 females) for check-up were randomly selected for *G6PD* gene variants detection, they were firstly tested for G6PD enzyme quantitative assay, and 8 cases (8%) were G6PD deficient. Then, all 100 cases were genotyped for *G6PD* mutations (Huang et al., 2023), 4 female specimens showed gene mutation but without the enzyme activity decrease, while 3 specimens (2 males and one female) showed a decrease in enzyme activity but without gene mutations. 10 cases were detected with *G6PD* mutations, including c.1376G>T heterozygote (4 cases), c.1388G>A heterozygote (1 case), c.1388G>A hemizygote (1 case), c.95A>G heterozygote (3 cases), and c.95A>G hemizygote (1 case). The allele frequency of *G6PD* variants in this population and in females was 10% and 14.3% (8/56), respectively.

## 4 Discussion

This study is the first molecular investigation of G6PD deficiency in the Yangjiang area of western Guangdong province in southern China. A total of 8459 subjects underwent the G6PD enzyme assay, and the prevalence of G6PD deficiency was found to be 7.54% in males and 3.42% in females in our study cohort. Comparing the prevalence of G6PD deficiency in Yangjiang with other areas in China, the frequency of G6PD deficiency was higher than the average frequency of 13 provinces in the southern region of the Yangtze River and was also higher than the average frequency of 8 provinces in the northern region of the Yangtze River (Liu et al., 2020). In all, the frequency of G6PD deficiency in Yangjiang region of our study cohort was only secondary to Guangxi province (*p* < 0.001) and Yunnan (*p* < 0.001) for the whole China (Fu et al., 2018; He et al., 2018). Furthermore, the prevalence was similar to that report from the neighboring Guangzhou (*p* = 0.025) (Tang et al., 2018) and Dongguan (*p* = 0.964) (Peng et al., 2015). The prevalence of G6PD deficiency in China was shown in Table 2.

**TABLE 2:** The incidence of G6PD deficiency in China.

Age	Province/City	Incidence	Sample number	References
Adult	Yangjiang	7.54% (M)	3314 (M)	This study
		3.42% (F)	5145 (F)	
Neonate	Wuhan	0.37% (M)	230152 (M)	Shen et al. (2022)
		0.05% (F)	200654 (F)	
Neonate	Ningbo	0.54%	82233	Pan et al. (2021)
Neonate	Xiamen	2.30%(M)	54,101 (M)	Wang et al. (2021)
		0.90% (F)	45,445 (F)	
Neonate	Shenzhen	3.54%	33,562	Gao et al. (2020)
Adult	Yunnan	8.63% (Dai)	1,530	He et al. (2018)
		5.91% (Jingpo)	372	
Neonate	Guangzhou	6.4% (M)	8 725 (M)	Tang et al. (2018)
		1.7% (F)	7 594 (F)	
Neonate	Sichuan	2.7% (M)	10,984(M)	Zhang et al. (2018)
		2.1% (F)	9,660 (F)	
Neonate	Guangxi	10.95% (M)	71511(M)	Fu et al. (2018)
		2.96% (F)	59124 (F)	
Neonate	Guizhou	1.94%	515	Huang et al. (2016)
Adult	Chaozhou	2.33% (M)	2013 (M)	Lin et al. (2016)
		4.3% (F)	2208 (F)	
Neonate-adult	Dongguan	7.60% (M)	3,885 (M)	Peng et al. (2015)
		3.00% (F)	12,579 (F)	
Neonate	Chaozhou	3.22% (M)	1365 (M)	Yang et al. (2015)
		2.03% (F)	1135 (F)	
under 7 years	Yunnan	2.5% (ethnic minority)	11759	Yao et al. (2013)
Neonate	North China	0.028%		Liu et al. (2020)
	South China	0.950%		
	Whole China	0.767%		
Preterm neonate	Chengdu	3.55‰	54025	Jiang et al. (2021)

Note: Male, M; female, F. dai and jingpo were minorities in china.

It has been reported that G6PD variants exhibit significant genetic polymorphisms in Chinese populations (Liu et al., 2017). The genotypes of G6PD variants show notable variations between the southern and northern regions of China, as well as among different ethnic groups (Yao et al., 2013; Han et al., 2016; Liu et al., 2020). Notably, c.1388G>A and c.1376G>T are prevalent throughout China, while c.487G>A, which was not found in our study cohort, is more common in northern China (Liu et al., 2017). In our study cohort, the three most common G6PD mutations were c.1388G > A, c.1376G > T, and c.95A > G, which accounted for 84% of the total disease alleles. Remarkably, the distribution of G6PD variants in Yangjiang was found to be similar to that in Guangzhou (Tang et al., 2018), Guangxi region (Fu et al., 2018), and Dongguan (Peng et al., 2015). The main genotypes observed in these regions were c.1388G>A, c.1376G>T, c.95A>G, and c.871G>A.

20 G6PD pathogenic variants were identified in our study, the polymorphism of c.1311C>T does not result in an amino acid change. In addition, c.1311 C>T/IVS-1193 T>C polymorphism had a relatively high frequency in the individuals with normal G6PD levels of Guangdong province (Lin et al., 2018). More interestingly, our study showed that c.871G>A was always linked with the c.1311C>T in this region. We hypothesized that the carriers with c.871G>A in Yangjiang might share the same ancestors. The polymorphism of 3′ UTR c.*+357A>G (rs1050757), which is often linked with c.1311C>T, may contribute to decreased G6PD (Amini and Ismail, 2013; Sirdah et al., 2017). However, we did not find this 3′ UTR polymorphism of G6PD gene in Guangdong province (Lin et al., 2016). 18 cases of c.1311C>T carriers with G6PD deficiency were identified in our study, and no other pathogenic variants was found, and there might be other mechanism to explain this conflict. The clinical significance of the c.1311C>T polymorphism remains unclear and requires further study. The exact reason why individuals with this polymorphism develop G6PD deficiency is yet to be determined.

G6PD deficiency presents a broad spectrum of clinical manifestations, ranging from asymptomatic cases to various hemolytic syndromes such as acute hemolytic anemia, favism, and neonatal hyperbilirubinemia. In severe cases, this deficiency can lead to kernicterus (Xu et al., 2021; Yang et al., 2023). Early identification of G6PD deficiency plays a critical role in effectively managing associated complications. The World Health Organization (WHO) recommends conducting G6PD screening for newborns if the male prevalence of deficiency is 3%–5% (WHO Working Group, 1989 19). However, in our study, the prevalence of G6PD deficiency in males in Yangjiang was found to be 7.54%, indicating the necessity of essential G6PD deficiency screening during the newborn period to alert parents about the potential risks of severe neonatal jaundice (Yang et al., 2023). Currently, G6PD deficiency is routinely included in newborn screening programs in southern China. Additionally, genetic counseling for couples may involve raising awareness and providing guidance on avoiding oxidative stressors to protect themselves from hemolytic triggers.

In our study cohort, the prevalence of hemizygous G6PD-deficient males in Yangjiang was found to be 7.54%. Theoretically, the corresponding prevalence in females can be predicted using the Hardy-Weinberg formula (Chan et al., 2008). Based on this formula, the prevalence of heterozygous G6PD-deficient females would be 14.51%, while 0.57% would represent homozygous females. However, our study only identified 3.42% of G6PD-deficient females. G6PD deficiency is classified as an X-linked recessive inborn error that primarily affects males, while heterozygous females may exhibit normal, intermediate, or deficient G6PD activity due to random X chromosome inactivation. Consequently, a significant proportion of heterozygous females with partial G6PD deficiency may have been overlooked in our study.

In our study, we randomly selected 100 healthy adults (44 males and 56 females) for check-up, and G6PD gene variant detection was performed. Among these individuals, 14.3% (8/56) of the females were identified as heterozygotes for G6PD variants, which is similar to the calculated prevalence of 14.51% in females.

## 5 Conclusion

In conclusion, this study provides valuable insights into the prevalence and molecular characterization of G6PD deficiency in the Yangjiang region, contributing to our understanding of this condition. Furthermore, offering genetic consultation to affected couples can effectively raise awareness about G6PD deficiency and neonatal jaundice. It also plays a crucial role in guiding individuals to avoid oxidative stressors and safeguarding their children from hemolytic triggers.

## Data Availability

The raw data supporting the conclusion of this article will be made available by the authors, without undue reservation.
